# Intraperitoneal Bupivacaine Effect on Postoperative Nausea and Vomiting Following Laparoscopic Cholecystectomy

**DOI:** 10.5812/aapm.16710

**Published:** 2014-07-08

**Authors:** Mitra Yari, Bahman Rooshani, Parisa Golfam, Nahid Nazari

**Affiliations:** 1 Department of Anesthesia, Imam Reza Hospital, Kermanshah University of Medical Sciences, Kermanshah, Iran; 2 Department of Nursing and Midwifery, Kermanshah University of Medical Sciences, Kermanshah, Iran

**Keywords:** Bupivacaine, Postoperative Nausea and Vomiting, Cholecystectomy, Laparoscopic

## Abstract

**Background:**

Postoperative nausea and vomiting (PONV) after laparoscopic cholecystectomy (LC) has multifactorial etiology. Pain and use of opioids are among the important factors.

**Objectives:**

The present study aimed to evaluate the efficacy of intrapritoneal (IP) injection of bupivacaine on PONV.

**Patients and Methods:**

This was a double-blind randomized clinical trial, conducted on 66 patients aged 20-60, ASA I or II, candidates for LC. Patients were randomly assigned to two groups. Bupivacaine group received 20 mL bupivacaine 0.25% in the gallbladder bed, before and after cholecystectomy and the control group did not. The incidence of nausea and postoperative pain intensity was measured with Visual analogue scale (VAS) at 1, 2, 3 and 4 hours after operation, at rest and when coughing and changing positions. Nausea and vomiting occurrence were assessed at the same times.

**Results:**

There were no demographic data differences between groups. No differences were found between the two groups, in terms of incidence of nausea and vomiting. Furthermore, both groups were similar with respect to opioid consumption, during four hours post-operation.

**Conclusions:**

Intraperitoneal bupivacaine administration at the beginning and end of laparoscopic cholecystectomy reduced only visceral and shoulder pains at the 4th postoperative hour, but had no effect on reducing neither PONV, nor opioid demand, during the first four postoperative hours.

## 1. Background

Laparoscopic cholecystectomy (LC) is an established, universally accepted procedure for cholelithiasis treatment, to decrease patient discomfort. This procedure is well tolerated by patients, however, an increased incidence of postoperative nausea and vomiting (PONV) predisposes patients to psychological distress increase and delayed recovery and discharge times (1, 2). Although the PONV has multifactorial etiology, one its main predisposing factors is postoperative pain (1). Opioids are routinely used for pain relief and control, but they increase the incidence of PONV by opioid receptor stimulation in the trigger zone (3). It seems that some surgical and anesthetic interventions can decrease postoperative pain and subsequently the opioid use, leading to a reduced level of PONV.

Pain following LC can be divided into three major groups:

1. Parietal pain caused by surgical incision;
2. Visceral pain originating from gallbladder bed and caused by visceral manipulation during the operation and finally
3. Shoulder pain secondary to distension of diaphragm by neuropraxia of phrenic nerve (4, 5).

Due to the different pathophysiological mechanisms of these pain components, different local anesthetic interventions have been developed to reduce postoperative pain, like peritoneal infiltration, instillation into the peritoneal cavity or subdiaphragmatic area, intraperitoneal spraying above the gallbladder and combined peritoneal-peritrocal ropivacaine (6-12). However, there is controversy about the characteristics of the pain components and also about the pain reducing the effects of intraperitoneal or peritrocal local anesthetics.

## 2. Objectives

The present study aims to evaluate the efficacy of intraperitoneal local anesthetic bupivacaine on PONV, after managing the visceral and shoulder pains. To avoid any confusion in different components of pain, we decided to eliminate parietal pain by injection bupivacaine into the abdominal wall, in both groups. So that we could fully concentrate on visceral and shoulder pain and to see if IP bupivacaine injection could affect the opioid demand by influencing visceral and shoulder pain.

Intra-abdominal dull pain that cannot exactly be located is considered as visceral pain, while the sharp pain felt in the abdominal wall is deemed as the parietal pain.

## 3. Patients and Methods

This was a double-blind, randomized clinical trial conducted on 66 patients of both sexes (aged 20 to 60 years), September 2010 to April 2011. All patients were registered for LC operation, according to the American Society of Anesthesiologists physical status I and II. All studies were performed in accordance with the ethical guidelines, set by the ethical committee of Faculty of Medical Sciences, Kermanshah University of Medical Sciences (Iran). The study was registered in Iranian Registry of Clinical Trials (IRCT13880112946N1). All patients were well informed of the study and all signed a written consent. The exclusion criteria were obesity (body mass index higher than 30 kg/m^2^), history of opioids abuse, antiemetic and steroids administrations within 24 hours prior to the surgery, history of motion sickness or PONV and patients with migraine whose surgery changed to open cholecystectomy. Patients were randomly assigned to two groups using a computer based randomization method. All patients received the same anesthetic agent and protocol. General anesthesia was induced by 0.2 µg/kg sufentanil, followed by 3-5 mg/kg thiopental and 0.6 mg/kg atracurium to facilitate tracheal intubation. Anesthesia was maintained with propofol infusion (200 µg/kg/minute) and injection of 0.1 µg/kg sufentanil, every 15 minutes.

Patients in bupivacaine group received 20 mL of bupivacaine in the gallbladder bed, after abdominal CO_2_ insufflation, as well as 20 mL of bupivacaine 0.25% in the gallbladder bed, after resection of gallbladder, whereas control group (n = 33) did not get such injections.

During operation, patients were placed in the reverse Trendelenburg position. Pneumoperitoneum was created with a closed Veress needle technique and LC was performed using four trocars, placed in the standard position. The gallbladder was retracted via a supraumbilical trocar port. During laparoscopy, intra-abdominal pressure was maintained at 12 mmHg. CO_2_ was carefully evacuated at the end of the operation by manual compression of the abdomen with open trocars. For elimination of the parietal pain and concentration on the visceral and shoulder pains, both groups received local administration of 5 mL bupivacaine 0.25% at incision of each trocar and different layers of abdomen. For reversal of muscle relaxation, 40 µg/kg neostigmine and 20 µg/kg atropine were administered and patients were transferred to the post anesthesia care unit (PACU) after tracheal extubation.

The incidence and severity of nausea and postoperative pain intensity at rest, when coughing and changing positions from supine to sitting were measured, using the visual analog scale (VAS) at one, two, three and four hours after the operation. The incidence of vomiting was evaluated by a “yes” or “no” question at the same time. At the first, second, third and fourth hours after surgery, when examining the intensity of pain and nausea, the patients were asked if they had vomiting. If the answer was positive, the frequency was recorded. All assessments were recorded by trained nurses who were blinded to the study and group assignments.

Patients could request for rescue analgesia and antiemetic at any time after operation. Fifty milligram tramadol and 10 mg metoclopramide was intravenously administered as a rescue analgesic and antiemetic, respectively (rescue antiemetic treatment in VAS > 3).

The statistical analysis was performed using SPSS package (SPSS, Chicago, IL, USA, version 16). For statistical analysis of the demographic data and for comparison of the two groups, Chi square, Mann-Whitney U-test and the student t-test analyses were performed.

## 4. Results

A total of 73 patients were assessed for eligibility, of which 66 patients were enrolled into the study. There were no differences among the groups with respect to patients' characteristics and operative data. Bupivacaine and control groups did not differ significantly in mean age (39.7 ± 11.3 vs. 42.39 ± 12 years; P = 0.368) and intraoperative opioid usage (32.61 ± 7.92 vs. 30.88 ± 7.20 microgram sufentanil; P = 0.381). Female patients comprised 97.1% of the bupivacaine group and 85.5% of the control group, indicating no significant difference in this regard (P = 0.166).

**Figure 1.**
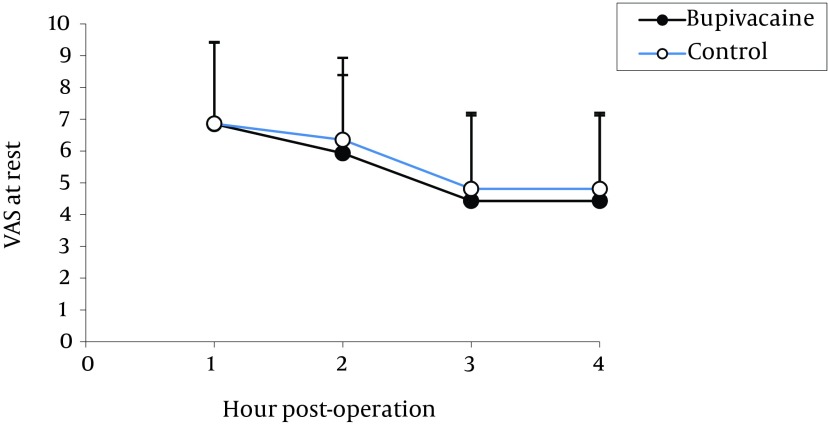
Comparison of Pain at Rest in the Two Groups

The means values for the pain VAS during resting, coughing and changing positions for four hours following operation are presented in Figure 1.

**Figure 2.**
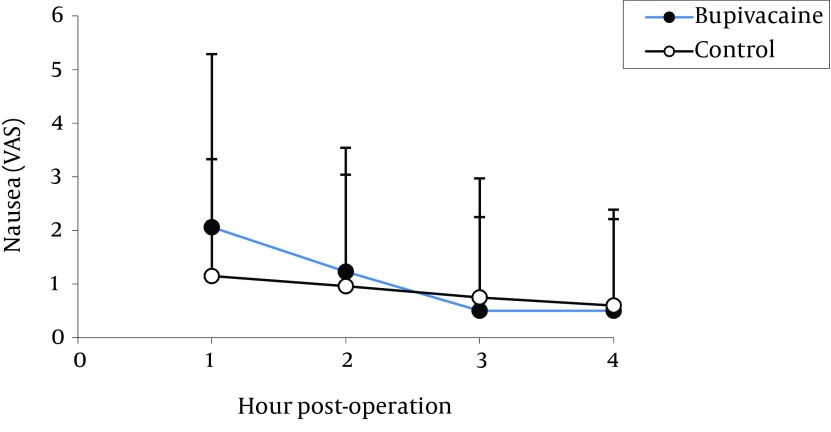
Comparison of Postoperative Nausea in Bupivacaine and Control Groups

**Figure 3.**
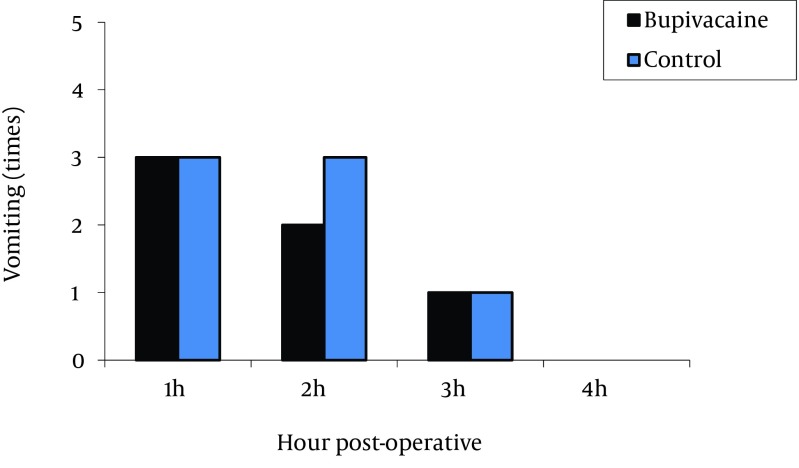
Comparison of Postoperative Vomiting in Bupivacaine and Control Groups

The highest pain levels were experienced during the first postoperative hour and these levels were gradually diminished with time. No significant difference was found between the two groups in terms of incidence of nausea (Figure 2) and vomiting (Figure 3). Vomiting occurred for six patients in the bupivacaine group and seven in the control group, who were then treated with 10 mg metoclopramide. Furthermore, both groups were similar with respect to opioid consumption, during the first postoperative four hours.

There was no significant difference in parietal pain between the two groups. Visceral and shoulder pains showed significant differences between the control and bupivacaine groups only in 4th post-operative hours. Although the viseral and shoulder pain intensity at the other time was lower in Bupivacaine group. That was not statistically significant (Table 1).

**Table 1.** Pain Component Data in the Two Groups

Post-Operative Pain	Control Group, (n = 33)	Bupivacaine Group, (n = 33)	P Value
**Shoulder pain, h**			
1	11	8	0.587
2	14	8	0.191
3	10	8	0.783
4	18	10	0.046
**Visceral Pain**			
1	22	23	0.792
2	24	19	0.301
3	23	23	0.999
4	22	14	0.048
**Parietal Pain**			
1	2	0	0.492
2	1	0	0.999
3	2	0	0.492
4	1	0	0.999

Variables related to analgesic consumption in the bupivacaine and control group is shown in Table 2.

**Table 2.** Comparison of Variables Related to Analgesic Consumption in the Bupivacaine and Control Group ^a^

variables	Control Group	Bupivacaine Group	P Value
**First analgesic request, h**	1.68 ± 0.98	1.27 ± 0.90	0.126
**Pain intensity based on VAS in first analgesic request**	7.40 ± 3.49	7.04 ± 3.24	0.711
**Mean dose of received analgesic after surgery, mg tramadol**	57.14 ± 29.55	64.1 ± 45.69	0.435

^a^ Data are presented as Mean ± SD.

## 5. Discussion

This randomized, double-blind, placebo-controlled trial aimed to investigate the effects of IP administration of bupivacaine on visceral and shoulder pain, as well as its effects on PONV after LC.

Our results showed that IP administration of 20 mL bupivacaine 0.25% in the gallbladder bed after abdominal CO_2_ insufflation and also after resection of gallbladder did not reduce the patient’s demand for extra opioids and had no effect on PONV.

The origin of pain and PONV, following LC is not entirely cleared, but it is probably multifactorial. Insufflation of CO_2_, resulting in stretching of the peritoneum, residual pneumoperitoneum after CO_2_ insufflation, peritoneum distension, diaphragm irritation and visceral organ irritation and manipulation have been reported to influence the severity of pain and PONV (5, 13).

Although, in several studies using local anesthetics in peritoneal cavity reduced postoperative pain and PONV, the use of different pain control techniques, including the application of intraperitoneal and/or peritrocar local anesthetics has still remained controversial (6, 12, 14).

Visceral and shoulder pain were significantly different at the 4th post-operative hour between the two groups. Some studies have shown that parietal pain can be blocked by infiltration of local anesthetic agents at incisions and visceral and shoulder pains by IP administration of local anesthetics (5-9). A meta-analysis performed by Boddy et al. showed the efficacy of IP local anesthetics in relieving post-laparoscopic cholecystectomy pain, without side effect of analgesic toxicity (15). Chou YJ et al. demonstrated that IP bupivacaine administration, is effective on the visceral pain reduction, both after trocar incision and at the end of the surgery whereas, with no efficient reduction in abdominal and right shoulder pain and opioid demand (16). In this study, parietal pain in both groups could be diminished through trocar bupivacaine administration to assess the effects of visceral and shoulder pains on PONV following LC. Our findings showed that IP administration of bupivacaine reduced both visceral and shoulder pains but had no effect on reduction of rescue analgesic. On the other hand, IP bupivacaine had not any impact on reduction of PONV and the incidence of nausea and vomiting were similar in both groups. Based on the findings of this study, it can be concluded that use of opioids has more effects than pain itself to increase PONV. It has been shown that opioid use increases the incidence of PONV by stimulation of opioid receptor in chemoreceptor trigger zone (CTZ) (3). Due to the presence of the blood-brain barrier at the CTZ, neurons at this region are stimulated by opioids present in the systemic circulation (17). Furthermore, opioid receptors are involved in reduction of bowel motility. This effect results in bowel distension, increased GI emptying time and constipation, leading to visceral chemoreceptors and mechanoreceptors stimulation. This effect is often responsible for nausea and vomiting in patients receiving opioid drugs (17-20).

Findings of this study demonstrated that 20 mL intraperitoneal bupivacaine 0.25% administration at the beginning and also at the end of the laparoscopic cholecystectomy process, reduced only visceral and shoulder pains at the 4th postoperative hour, but had no effect on reducing neither PONV, nor opioid requirement, during the first four postoperative hours. However, further studies are needed on larger populations with different doses and concentrations of bupivacaine and also combination of bupivacaine and other analgesic drugs, to gain the maximum benefits of intraperitoneal analgesic in postoperative pain treatment.
